# Metabolic–epigenetic imprinting of macrophages in endometriosis: a working model for lesion persistence and treatment

**DOI:** 10.3389/fimmu.2026.1940736

**Published:** 2026-09-24

**Authors:** Yaru Wang, Wanqiu Xia, Xueting Su, Lei Fang

**Affiliations:** Department of Obstetrics and Gynecology, The Second Affiliated Hospital of Harbin Medical University, Harbin, China

**Keywords:** endometriosis, histone lactylation, immunometabolism, macrophage memory, TET3, trained immunity

## Abstract

Endometriosis is an estrogen-dependent inflammatory disorder that often persists or returns even after visible lesions have been removed. The behavior of peritoneal macrophages may help explain this clinical durability. These cells participate in lesion implantation, vascularization, fibrosis, defective immune clearance, and pain, but it is uncertain why disease-supporting phenotypes are maintained. We argue that repeated exposure to the endometriotic niche may leave macrophages with a metabolically conditioned epigenetic memory rather than only a short-lived polarization state. Lactate, succinate, iron, oxysterols, and glutamine-derived metabolites could alter cellular metabolism while also providing substrates, cofactors, or inhibitors for chromatin-modifying enzymes. Histone lactylation and TET/JmjC-regulated methylation states are plausible molecular records of this exposure, although neither has yet been followed from metabolite to persistent mark and macrophage function in human disease. At present, the most direct endometriosis-specific support comes from a TGF-β1/CCL2-induced TET3 program associated with let-7 repression, inflammatory cytokine production, and macrophage survival. Other proposed circuits, including lactate–lactylation coupling and macrophage–stromal exchange of glycolytic signals, remain incomplete. This distinction matters: established findings support parts of the model, but not yet the entire causal chain. A memory state should be experimentally identifiable by its persistence after stimulus withdrawal, transferability, metabolic dependence, and reversibility. If experiments satisfy these criteria, the retained macrophage state would become a plausible non-hormonal target. The relevant test would be whether changing that state produces a lasting loss of lesion-supporting function instead of a brief reduction in downstream inflammation.

## Introduction

Approximately one in ten women of reproductive age are affected by endometriosis, in which endometrium-like tissue occurs outside the uterine cavity (1). It is a major cause of pelvic pain and infertility, yet its etiology remains uncertain. Analgesics, hormonal suppression, and surgery are the main treatment options, and none is curative (2). Each also has practical limits: lesions may recur after conservative surgery, while hormonal treatment is unsuitable when pregnancy is being attempted (2). Durable care that preserves fertility has therefore been slow to emerge (3). These clinical observations raise a further possibility. Removing visible implants may not normalize all of the biology that supported them. The peritoneal compartment could retain changes that favor renewed lesion growth after apparent clearance, although the nature and duration of such a residual state are unknown.

Macrophages are plausible participants in this residual biology. They are abundant in the endometriotic peritoneal compartment and are repeatedly recruited to ectopic tissue, where they contribute to attachment, angiogenesis, fibrosis, and nociceptive sensitization (4). Human single-cell data are consistent with these functions. An integrated endometrial atlas assigned part of the genetic risk for endometriosis to macrophages and decidualized stromal cells (5), and macrophages within lesions are enriched for immunosuppressive and tissue-repair programs that can favor ectopic tissue survival (6). These findings justify examining macrophages as both disease contributors and therapeutic candidates, without implying that they act independently of stromal, epithelial, or other immune cells.

Two lines of evidence have developed largely in parallel. First, endometriotic lesions and their associated macrophage niches show evidence of metabolic rewiring, with enhanced glycolysis in the lesion compartment and metabolically altered immune states in the peritoneal environment (7). Second, metabolites can do more than provide bioenergetic currency; in macrophage systems, lactate can supply histone lysine lactylation and thereby link glycolytic output to transcriptional regulation (8). These observations converge on the concept of trained immunity, in which epigenetic and metabolic rewiring confers a lasting functional memory on innate immune cells (9). Critically, histone lactylation has now been shown to persist for months *in vivo* and to mark genes poised for enhanced secondary responses, linking a metabolite-derived mark to durable innate memory (10). The disease relevance of macrophage memory is supported in endometriosis by experiments in which reprogramming peritoneal macrophages toward opposing trained states bidirectionally controls lesion growth, with pro-inflammatory training aggravating disease and low-dose endotoxin tolerization suppressing it through interleukin-10 (11).

What has been missing is a framework that unites these strands. Metabolic reprogramming, epigenetic remodeling, and innate immune memory are typically discussed as separate features of endometriosis-associated macrophages, and existing macrophage-focused reviews have emphasized polarization or metabolism without invoking durable memory as an organizing principle (7). An endometriosis-supported TET3-dependent pathogenic program, induced by TGF-β1 and CCL2 and associated with let-7 suppression, IL-1β/IL-6-associated inflammation, and enhanced macrophage survival, provides disease-specific evidence for pathogenic macrophage reprogramming, but its persistence after stimulus withdrawal and metabolic basis are unproven (12). Interpreting this and related findings through the lens of trained immunity suggests that endometriosis-associated macrophages may acquire an imprinted disease-promoting state rather than representing only transient polarization.

We therefore propose a unifying framework in which metabolic, inflammatory, and endocrine inputs from the endometriotic niche may be encoded through chromatin-regulatory mechanisms into a self-reinforcing pathogenic memory state in endometriosis-associated macrophages (EAMs) (**Figure 1**). This framework distinguishes endometriosis-supported evidence, exemplified by the TET3 feed-forward circuit, from proposed or partly extrapolated lactate–lactylation and trans-cellular metabolic loops. We first outline the metabolite composition of the endometriotic niche and the metabolic rewiring it may impose on resident macrophages. We then examine how metabolites can act as cofactors and substrates for chromatin-modifying enzymes, and how such marks might stabilize a disease-promoting phenotype that behaves as innate immune memory. We next consider how the cyclical, estrogen-dominated hormonal environment of the disease might periodically modulate this memory. Finally, we consider how the memory might be erased or reprogrammed, whether through metabolic inhibitors, epigenetic modulators such as the TET3 degrader Bobcat339, or tolerizing regimens. This last step positions the reversibility of macrophage memory as a candidate non-hormonal strategy to limit lesion persistence and potential recurrence. We stress that this is a hypothesis-and-theory framework rather than a settled mechanism: its purpose is to organize existing evidence into falsifiable predictions, and we are explicit throughout about which links are supported by direct endometriosis data, which are partial, and which are extrapolated from other systems.

**Figure 1:**
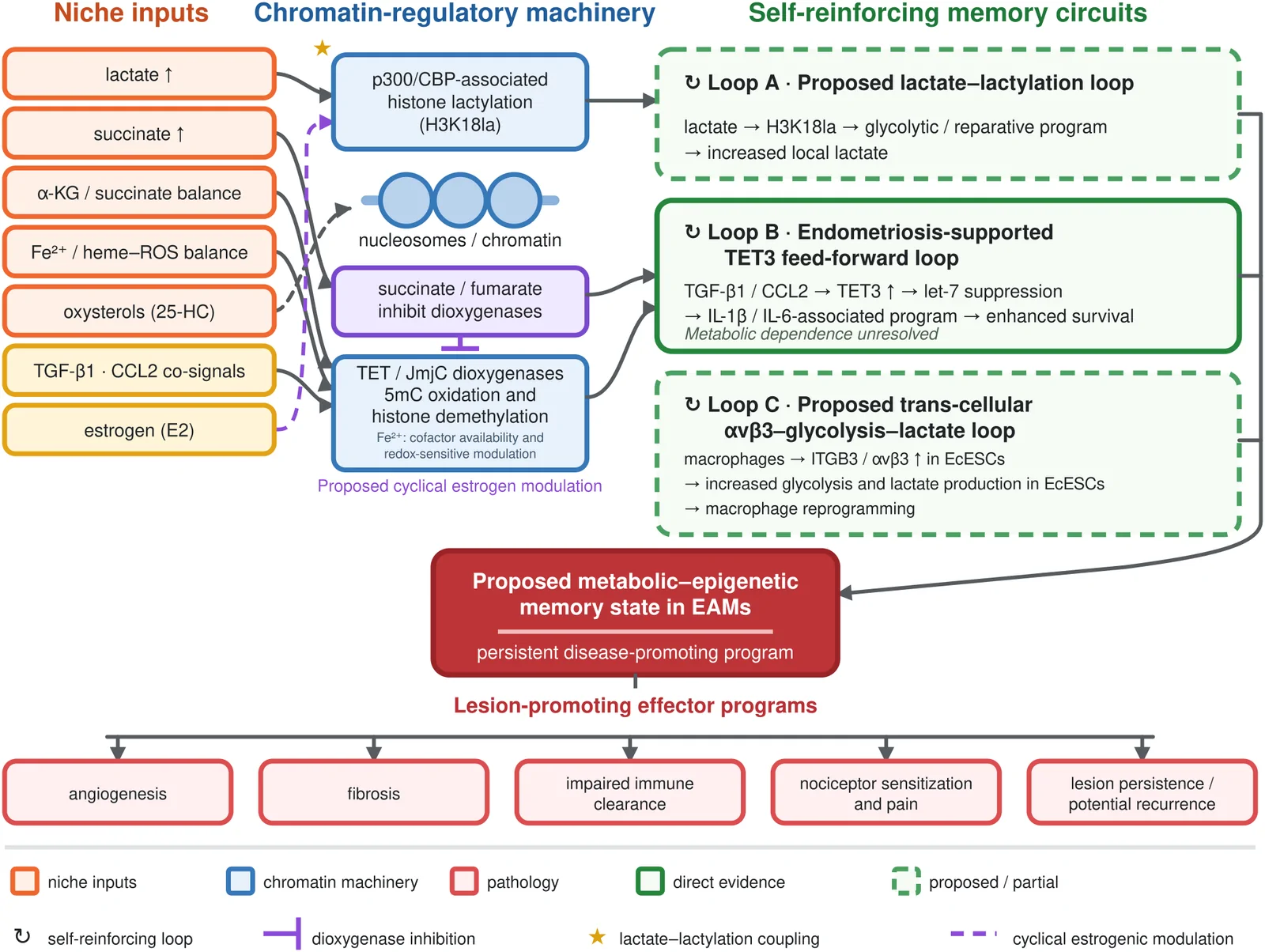
Proposed metabolic–epigenetic circuits sustaining pathogenic macrophage states in endometriosis. Niche inputs converge on chromatin-regulatory machinery in endometriosis-associated macrophages (EAMs). Lactate may promote H3K18 lactylation (H3K18la). α-Ketoglutarate (α-KG) supplies a co-substrate, while Fe²^+^ availability and redox context may modulate TET/JmjC dioxygenase activity; succinate and fumarate inhibit these enzymes. Oxysterols, including 25-HC, are included as candidate inputs that may bias macrophages toward immunoregulatory or reparative states; their contribution to EAM memory remains unproven. Loop A proposes a lactate–H3K18la circuit. Loop B depicts the endometriosis-supported TET3 program: TGF-β1/CCL2-induced TET3 is associated with let-7 suppression, IL-1β/IL-6 output, and macrophage survival. These components are supported in endometriosis, but the complete autocrine loop and its metabolic dependence remain unresolved. Loop C proposes that macrophage-dependent ITGB3/αvβ3 induction in ectopic endometrial stromal cells (EcESCs) increases glycolysis and lactate production in EcESCs, which may feed back on macrophages. Together, these circuits may stabilize a state linked to angiogenesis, fibrosis, impaired immune clearance, pain, and lesion persistence or recurrence. Gray arrows denote mechanistic relationships; solid and dashed green borders denote direct endometriosis-specific evidence and proposed or partly supported mechanisms, respectively; the purple dashed arrow denotes proposed cyclical estrogen modulation. Abbreviations: EAMs, endometriosis-associated macrophages; EcESCs, ectopic endometrial stromal cells; α-KG, α-ketoglutarate; 25-HC, 25-hydroxycholesterol; E2, 17β-estradiol; ITGB3, integrin subunit beta 3.

## Endometriosis-associated macrophages: origin, heterogeneity, and pathogenic roles

Macrophages are the dominant immune population of the endometriotic peritoneal cavity, and their identity in this setting is best understood as a mixture of ontogenetically distinct cells shaped by a shared pathological environment. Two sources converge on the lesion: long-lived tissue-resident macrophages seeded before birth, and monocyte-derived cells recruited continuously from the circulation in response to chemokines such as CCL2 (4). Retrograde menstruation delivers not only ectopic endometrial fragments but also erythrocytes, cellular debris, and inflammatory mediators. The macrophages that respond to this insult acquire functional states that favor, rather than resolve, ectopic tissue survival, to the extent that endometriosis has been described as a disease of the macrophage (13). This altered immune milieu extends to the eutopic endometrium, where the immune microenvironment is itself perturbed in affected women (14). Endometriosis is increasingly understood as an immune-mediated disorder in which impaired clearance and a permissive peritoneal environment allow ectopic tissue to establish (15).

Single-cell transcriptomics has replaced the binary M1/M2 scheme with a more granular view of macrophage heterogeneity in endometriosis. An integrated single-cell reference atlas of the human endometrium was built from more than half a million cells and mapped against the largest endometriosis genome-wide association meta-analysis. It localized disease-associated genetic risk to macrophages and to subsets of decidualized stromal cells. These data prioritize macrophages as candidate disease-relevant cells rather than establishing causal primacy (5). Atlases of ectopic and eutopic tissue have resolved discrete lesional subsets, including C1q-high, CD163+CD206+ macrophages with an immunosuppressive signature (16). Endometriosis-associated macrophages skew toward immunosuppressive, pro-repair states, and the reparative bias they acquire prefigures the tumor-associated macrophage programs seen in cancer (17). Consistent single-cell and integrated analyses report an enrichment of pro-repair, M2-skewed cells across disease progression (18). Spatial transcriptomics now places these subsets *in situ*. Signaling from the lesion epithelium, including complement C3, actively drives a pro-repair macrophage phenotype (19), and single-cell and spatial profiling of the lesion niche resolves the interactions that sustain its growth (20). Together, these datasets point to several macrophage states rather than one uniform pathogenic population. As a group, however, they are shifted toward angiogenic and tissue-remodeling phenotypes with limited clearance capacity.

The functional consequences of this skewing are well documented. Lesion-associated macrophages promote the attachment and vascularization of ectopic implants, drive fibrotic remodeling, and sensitize nociceptive nerves that underlie endometriosis-associated pain (6, 21). The causal weight of these roles is established by depletion experiments. Removing CD206+ macrophages reduces lesion angiogenesis and cuts lesional VEGFA and TGF-β1, identifying this M2 subset as an accelerator of disease (22), while macrophage-derived insulin-like growth factor-1 directly sensitizes nerves to generate pain (23). This pro-repair bias is enforced hormonally as well. Reviews of estrogen–macrophage interactions indicate that estrogen promotes macrophage recruitment and M2-skewed signaling and may attenuate phagocytic and pro-inflammatory functions (24). In a co-culture model, combined 17β-estradiol and TCDD synergistically promoted M2 differentiation through STAT3 and p38-MAPK signaling (25), and interleukin-17A reinforces M2 skewing in the peritoneal cavity (26). These cells also handle the iron burden imposed by cyclic erythrocyte breakdown. Excessive erythrophagocytosis may load peritoneal macrophages with iron and heme and, when detoxification capacity is exceeded, may contribute to M2/angiogenic skewing or ferroptotic injury; whether this directly impairs lesion clearance remains unresolved (27, 28). The result is a self-defeating loop, in which the cells meant to clear ectopic tissue instead provide the growth factors and permissive immune context that let it persist (29).

Functional reprogramming experiments indicate that at least some macrophage states can persist beyond the signal that initially induced them. In the study by Jeljeli and colleagues, peritoneal macrophages driven toward opposing trained states altered lesion growth in opposite directions, and transferred cells retained those effects in untreated recipients (11). Transfer to a naive host is especially informative because it argues against a purely moment-to-moment response to the local cytokine milieu. The immunosuppressive and tissue-repair bias seen in single-cell datasets may therefore reflect a cellular imprint in some macrophage populations rather than continuous instruction alone. It should not yet be assumed, however, that every disease-associated subset is stably programmed.

These observations shift the practical question from how to block individual macrophage-activating signals to why a disease-supporting macrophage state persists. Depletion and repolarization reduce disease in preclinical models, but their interpretation is limited by uncertainty over which subsets are harmful, which are protective, and how cellular origin affects plasticity. A mechanistic account must therefore explain how the pro-repair bias survives signals that would ordinarily promote resolution. It must also establish whether that durability is recorded in chromatin and whether the altered metabolism of the lesion provides the relevant input. The first part of that problem is the chemical environment encountered by peritoneal and lesion-associated macrophages.

## Metabolic features of the endometriotic niche

Peritoneal fluid is the immediate chemical environment of superficial lesions and many of the macrophages considered here. Rather than reflecting systemic metabolism alone, it integrates products released by ectopic tissue, mesothelium, recruited immune cells, and recurrent bleeding. Metabolomic studies describe increased glycolytic activity together with changes in amino-acid and lipid metabolism against a background of iron exposure (30). These measurements are relevant because macrophages continuously import metabolites and respond to inflammatory mediators in the same compartment. Defining what is present locally is therefore necessary before asking whether repeated metabolic exposure can leave a durable macrophage phenotype.

Lactate is one of the more reproducible findings in studies of the endometriotic peritoneal environment. Horne and colleagues reported reduced mitochondrial respiration and greater aerobic glycolysis in ectopic lesions and endometriosis-derived mesothelial cells, with lactate released into the surrounding fluid (31). Young et al. linked this response to TGF-β1. In primary peritoneal and mesothelial cells, TGF-β1 increased HIF1A, SLC2A1, PDK1, and LDHA expression and raised lactate production; patient samples showed a parallel association between TGF-β1 and fluid lactate (32). Lactate can also enhance ectopic-cell growth and invasion (7). These observations establish biological activity in the lesion compartment, but they do not establish the macrophage step proposed here. In particular, no tracing experiment has shown that extracellular lactate becomes a long-lasting lactylation mark in EAMs (33).

Peritoneal succinate has also been measured as more than a by-product of metabolism. Tian et al. found higher concentrations in patients, an association with disease severity, and SUCNR1-dependent effects on adhesion and implantation of ectopic stromal cells (34). Those experiments support a direct action on ectopic tissue. The macrophage interpretation is less straightforward. Succinate can produce inflammatory or immunoregulatory responses in macrophage systems (35), and its effect varies with dose, exposure time, and cellular state. For this reason, the presence of succinate in the cavity does not by itself predict an EAM phenotype. Its role has to be tested in macrophages under conditions that approximate the concentrations and timing found in endometriosis.

Glutamine is a more tentative component of the proposed niche. The recent multicenter metabolomic study emphasized lipid-related signatures and did not demonstrate consistent glutamine depletion in peritoneal fluid (36). Its relevance comes instead from established macrophage biochemistry: glutaminolysis supplies α-ketoglutarate to the TCA cycle, and related pathway flux can generate fumarate. The former supports α-ketoglutarate-dependent chromatin enzymes, whereas the latter can inhibit demethylases (37, 38). Whether either mechanism operates in EAMs cannot be inferred from these general findings. Direct measurements of glutamine availability and pathway flux in the relevant peritoneal macrophage subsets are still needed.

Iron and heme define a fourth axis, one nearly unique to endometriosis among inflammatory diseases. Cyclic retrograde menstruation floods the cavity with erythrocytes whose breakdown releases hemoglobin, heme, and free iron, and peritoneal macrophages are positioned to clear this load (27, 28). Lesional and peritoneal-fluid macrophages upregulate heme-handling and iron-storage machinery such as HMOX1 and ferritin, alongside iron accumulation in lesions. When heme-detoxification capacity is overwhelmed, HMOX1 may shift from a protective to a pro-oxidant role and promote ferroptosis; in endometriosis, the consequences for lesion survival and macrophage fate remain context-dependent (27, 28). In parallel, iron overload in peritoneal fluid from patients with endometriosis can induce HMOX1-mediated ferroptosis in early embryos, linking the same niche exposure to infertility (39). Iron overload has been proposed to be directly cytotoxic to peritoneal macrophages, in part through nitric-oxide-driven apoptosis (40). It may also bias macrophage polarization toward a reparative, angiogenic phenotype, coupling repeated bleeding to a candidate immunometabolic output (27, 28).

PGE2 provides a link between lipid metabolism and the endocrine environment. Ectopic endometrial cells produce PGE2 through cyclooxygenase-2, and macrophage migration inhibitory factor increases this production in ectopic cells (41). PGE2 can then stimulate aromatase and local estrogen synthesis (42). The quantitative contribution of peritoneal macrophages to this pool is not known. A separate line of metabolomic evidence shows altered glycerophospholipids and acylcarnitines, including changes in phosphatidylcholine species (43). Oxidized cholesterol metabolites are relevant because they directly influence macrophages. For example, 25-hydroxycholesterol can promote an immunosuppressive state through lysosomal and LXR-dependent pathways (44), but it can also enhance TLR4-driven inflammation in lipid-loaded cells (45). In other settings, the same metabolite supports efferocytosis and resolution (46). Endometriosis studies have not yet resolved lesion-level oxysterol profiles or demonstrated cholesterol-loaded EAMs (43, 47). The proposed oxysterol mechanism therefore remains separate from the lipid changes that have actually been measured in patients.

Available measurements describe a chemically mixed peritoneal compartment: glycolytic products and succinate are increased in some cohorts, lipid and amino-acid profiles are altered, and bleeding repeatedly adds iron. A macrophage at a lesion boundary encounters these factors together. The dichloroacetate studies show that at least one component of this environment can be modified pharmacologically, because treatment reduced peritoneal lactate while correcting lesion metabolism (31). Bulk-fluid measurements nevertheless have limited spatial and temporal resolution. They cannot determine the dose experienced by a particular macrophage or how that dose changes over a cycle. The crucial experiment has also not been done: a measured niche metabolite has yet to be causally linked to a persistent chromatin change in a defined EAM subset.

## Metabolic adaptation of macrophages within endometriotic lesions

Exposure to an altered niche does not by itself specify macrophage behavior; the cells also change how they use metabolic pathways. Work in other macrophage systems has shown that inflammatory activation is often accompanied by increased aerobic glycolysis, whereas oxidative phosphorylation and fatty-acid oxidation are more compatible with reparative programs (48). This contrast emerged from studies connecting PGC-1β-dependent oxidative metabolism with reduced inflammatory activity (49) and lysosomal lipolysis with alternative activation (50). Later network analyses showed that polarization is supported by several partially independent metabolic modules rather than a simple two-pathway switch (51). That qualification is especially important in endometriosis. EAMs are exposed simultaneously to lactate, hypoxia, lipids, iron, cytokines, and ovarian steroids, so their metabolism is more likely to be mixed and subset-specific than uniformly glycolytic or oxidative.

Evidence for glycolytic reprogramming is strongest at the level of the lesion, not yet within purified macrophage subsets. Endometriotic tissue shows increased aerobic glycolysis and reduced mitochondrial oxidative phosphorylation in a lactate-rich, TGF-β1-associated environment (7). Integrated transcriptomic analyses have also identified metabolic-reprogramming genes that correlate with immune-cell composition, but correlations and expression scores do not measure macrophage-intrinsic flux *in situ* (52). A stromal co-culture circuit provides more direct evidence of communication between cell types: macrophages increase ITGB3/αvβ3 in ectopic endometrial stromal cells, which raises glycolysis and lactate production; lactate then supports stromal proliferation, migration, and invasion (53). The macrophage-to-stroma arm is supported experimentally. Whether stromal lactate returns to impose a stable program on macrophages remains unresolved.

Interrupted TCA-cycle flux can also generate signals with immunological consequences. In inflammatory macrophages, accumulated succinate stabilizes HIF-1α, sustaining glycolytic gene expression and IL-1β transcription (54, 55). A different interruption produces itaconate through ACOD1/IRG1. Itaconate inhibits succinate dehydrogenase, limits excessive inflammation, and activates NRF2-dependent antioxidant responses (56, 57); this biology has prompted development of itaconate-related anti-inflammatory approaches (58). Endometriotic lesions provide two inputs that could engage these pathways—extracellular succinate and local hypoxia—but direct measurements of TCA flux in EAMs are still lacking. The proposed connection is therefore mechanistically plausible rather than disease-validated.

Amino acid and lipid metabolism supply candidate routes into the reparative arm of macrophage reprogramming. Glutaminolysis feeds the TCA cycle and generates α-ketoglutarate, which promotes anti-inflammatory, M2-like polarization and endotoxin tolerance while restraining pro-inflammatory NF-κB signaling in macrophage models (37). Interleukin-10, which mediates the disease-limiting effect of tolerized macrophages in endometriosis, can enforce oxidative metabolism by inhibiting glycolysis and promoting mitophagy of damaged mitochondria (11, 59). Cholesterol and its oxidized derivatives add a further layer: LXR activation by oxysterols drives cholesterol efflux and an anti-inflammatory, arginase-expressing phenotype, integrating lipid handling into macrophage polarization (60). These pathways provide plausible mechanisms by which the lipid- and amino-acid-remodeled niche could bias EAMs toward the immunosuppressive, pro-repair states observed by single-cell studies, but their relative contribution in endometriosis remains to be quantified.

Iron metabolism is the axis that most clearly ties a disease-specific input to a macrophage metabolic fate. Erythrophagocytosis loads macrophages with iron, and the resulting oxidative stress, if not buffered by heme oxygenase and ferritin, may promote lipid peroxidation and ferroptosis and may bias surviving cells toward an angiogenic, reparative output (27, 28). Macrophage-generated reactive oxygen species are themselves pro-angiogenic and accelerate early lesion development (61), providing a separate redox route to neovascularization. Iron is also a cofactor for α-ketoglutarate-dependent dioxygenases, but in the endometriotic niche its effect is likely to be redox- and context-dependent rather than simply activating. The iron status of an EAM should therefore be considered a potential modifier of chromatin-regulatory enzymes, a point developed in the next section.

Across these pathways, the metabolic reprogramming of EAMs is defined less by a single dominant route than by the convergence of several pressures: exposure to a glycolytic and lactate-rich niche, oxidative competence compatible with a reparative phenotype, and iron and lipid handling that can both stress and instruct the cell. The translational appeal of this picture is that metabolic nodes are pharmacologically accessible; normalizing lesion metabolism with the pyruvate dehydrogenase kinase inhibitor dichloroacetate lowers peritoneal lactate and constrains disease in models (31). The central unresolved question, however, is one of durability. Metabolic states are, in principle, rapidly reversible, yet the macrophage phenotypes of endometriosis persist and can be transferred to naive hosts. Explaining that persistence requires a mechanism that could outlast the initiating metabolic signal. The following section examines candidate chromatin-regulatory mechanisms through which metabolites may contribute to such durability.

## Metabolic inputs and epigenetic remodeling in macrophages

The persistence that eludes a purely metabolic explanation becomes plausible once metabolism is recognized as an input to the epigenome. The enzymes that write and erase chromatin marks depend on intermediary metabolites as their substrates and cofactors, so metabolite availability can be transduced into the activity of chromatin-modifying enzymes and the transcriptional accessibility of specific loci (62). This principle, that intermediary metabolism and the epigenome are continuous rather than separate, is now a central theme of trained immunity and of macrophage memory more broadly (63, 64). The dependency is what allows a transient metabolic environment to leave a stable mark: a chromatin modification, once deposited, can outlast the metabolite that licensed it. For EAMs, whose niche is enriched for lactate, succinate, altered α-ketoglutarate balance, iron, and lipid mediators, several inputs plausibly map onto specific arms of chromatin-regulatory machinery.

Lactate is the paradigmatic example and the most directly relevant to the endometriotic niche. Lactate serves as the precursor for histone lysine lactylation, a modification in which lactyl groups are deposited on histone tails and can stimulate transcription from the underlying chromatin (8). In macrophages, lactylation was first characterized during polarization, where it accumulates on histone H3 lysine 18 (H3K18la) at the promoters of homeostatic and reparative genes, providing a mechanism by which glycolytic output can be converted into a specific transcriptional program rather than a generic one (8, 65, 66). Genome-wide, H3K18la preferentially marks tissue-specific active enhancers, extending its reach beyond promoters to the regulatory elements that define cell state (67). p300/CBP-associated mechanisms can write lactylation, and deposition can scale with intracellular lactate (8), making the lactate-rich endometriotic cavity a plausible substrate source for macrophage lactylation rather than a demonstrated EAM lactylation reservoir. Developmental systems likewise show that histone lactylation can couple cellular metabolism to gene-regulatory networks (68). The significance of lactylation is that it links the most reproducibly elevated metabolite of the niche to a transcription-activating chromatin state that is testable in EAMs.

The α-ketoglutarate-dependent dioxygenases connect amino acid metabolism, iron status, and redox balance to the erasure of methylation marks. α-Ketoglutarate, generated by glutaminolysis, is an obligatory cofactor for both the JmjC-domain histone demethylases and the ten-eleven translocation (TET) dioxygenases, with iron and oxygen as additional requirements (69). Through JMJD3-mediated demethylation of H3K27me3, α-ketoglutarate relieves repression of anti-inflammatory genes and promotes M2-like polarization and endotoxin tolerance in macrophage models (37). TET outputs are non-redundant rather than uniformly pro-inflammatory. Macrophage TET2 frequently restrains late inflammatory gene expression, partly through catalytic-independent recruitment of HDAC2, whereas TET3 promotes IL-1β/IL-6-associated programs and survival in endometriosis-associated pathogenic macrophages (12). These divergent functions caution against treating TET activity as a single inflammatory axis. Because the niche may alter α-ketoglutarate availability, succinate balance, iron loading, and redox state, it could modulate these enzymes, but direct evidence that α-ketoglutarate or iron availability drives TET/JmjC activity in EAMs remains lacking. Disease-specific evidence that DNA methylation machinery is altered in endometriosis is accumulating. DNMT enzymes are aberrantly expressed in lesions and DNA methylation is broadly dysregulated (70). Hydroxymethylation in endometrial biology remains incompletely mapped (71). In ovarian endometrioma, ectopic tissue has been reported to show increased 5-hydroxymethylcytosine and TET1, and hypoxia-induced TET1 has been implicated in epithelial-mesenchymal transition (72).

Succinate and fumarate act as the counterweights, inhibiting the same dioxygenases that α-ketoglutarate fuels. Both metabolites accumulate when the TCA cycle is remodeled, and both competitively inhibit α-ketoglutarate-dependent demethylases; fumarate, in particular, inhibits KDM5 histone demethylases and can, on its own, install an epigenetic program resembling that of trained immunity (38). The ratio of α-ketoglutarate to succinate therefore functions as a metabolic rheostat on chromatin, and the succinate-rich endometriotic niche is positioned to bias it toward retention of methylation marks. This antagonism illustrates a general principle: the epigenetic consequence of the niche is set not by any single metabolite but by the balance among them, and that balance is itself a product of the disease’s metabolic reprogramming.

Durable metabolite-linked chromatin changes are not merely theoretical. Genome-wide analyses of trained and tolerized macrophages show that β-glucan and related stimuli leave distinct promoter and enhancer profiles that remain after the initiating exposure has ended (73). Akt–mTOR–HIF-1α-dependent glycolysis is required while trained immunity is established, although it is not yet clear whether the same flux must continue to maintain the state (74). Histone lactylation offers a particularly relevant example: *in vivo*, it can persist for months at genes primed for stronger secondary responses (10). A metabolite-derived histone mark can therefore participate in long-lived innate immune memory, even though equivalent persistence has not yet been shown in EAMs.

Mechanistically, several entry points are plausible. Lactate may change histone lactylation; α-ketoglutarate, succinate, and fumarate may shift dioxygenase or demethylase activity; and iron status may affect those enzymes through cofactor availability and redox stress. A chromatin mark that decays slowly could extend the effect of a short metabolic exposure. At present, these are candidate links. Most of the underlying biochemistry was established in model macrophages or in other diseases, and endometriosis studies have not measured a candidate metabolite and its proposed chromatin mark in the same EAMs. Consequently, the coupling described below is presented as a working hypothesis whose individual steps require direct testing.

## Working hypothesis: can macrophages store metabolic exposure?

Here, memory is used in a strict experimental sense. A macrophage would have to retain altered function after removal from the initial endometriotic stimulus; a current activation profile alone would not qualify. In trained immunity, metabolic remodeling during induction is accompanied by chromatin changes that alter responses to a later challenge (9). The same process can be harmful when it preserves an inappropriate response in sterile inflammatory disease (75). Applied to endometriosis, the proposal is not that every EAM is trained. Rather, a stable cellular record may account for the part of the macrophage phenotype that cannot be explained by ongoing exposure alone.

The most relevant disease experiment was reported by Jeljeli et al. (11). Bacille Calmette–Guérin-conditioned peritoneal macrophages increased lesion growth, whereas repeated low-dose endotoxin produced an IL-10-dependent tolerant phenotype that limited disease. Transferring the tolerized cells to untreated recipients preserved the protective effect. Because the cells entered a new host, continuous instruction by their original environment cannot fully explain the result. The two conditioning regimens were also associated with different histone-methylation patterns at cytokine loci and with different lactate outputs. This study shows that an experimentally imposed macrophage memory can influence endometriosis. It does not show that lactate, succinate, iron, or another endogenous lesion metabolite creates the same memory spontaneously.

The endometriotic niche is well positioned to install or maintain a disease-promoting version of this memory, because it supplies candidate training stimuli endogenously and continuously. Whereas experimental trained immunity relies on defined microbial ligands, the endometriotic cavity presents a chronic mixture of lactate, succinate, iron, oxysterols, cytokines, and endocrine signals, each of which could engage the metabolic and epigenetic machinery described earlier. The persistence of at least one metabolite-derived mark is not merely assumed: histone lactylation persists for months *in vivo* and identifies genes poised for enhanced secondary responses (10). Whether the lactate-rich endometriotic niche imposes an equivalent persistent H3K18la program in EAMs remains a central test of the model.

The clearest disease-specific instance of pathogenic macrophage reprogramming is the TET3 program; persistence after stimulus withdrawal remains unproven. In endometriosis lesions, microenvironmental TGF-β1 and CCL2 drive TET3 overexpression and generate a distinct pathogenic macrophage population (12). TGF-β signaling is itself elevated across the disease and repeatedly implicated in lesion establishment, making it a credible upstream driver of this program (76). The endometriosis study supports TGF-β1/CCL2-induced TET3 expression and TET3-dependent let-7 suppression, IL-1β/IL-6-associated inflammatory output, and macrophage survival (12). A subsequent cross-disease study showed that TET3 can increase TGF-β1 and CCL2 expression, supporting a potential autocrine feed-forward loop in pathogenic macrophages (77). Whether the complete feedback circuit operates within human endometriotic lesions remains to be established. These macrophages also become dependent on elevated TET3 for survival and undergo apoptosis when it is restored to baseline (12). This provides disease-specific evidence for a TET3-dependent pathogenic program in EAMs. Its catalytic mechanism and metabolic dependence remain unresolved, so it should not be treated as proof that lactate, α-ketoglutarate, or succinate directly drives TET3 in endometriosis.

**Figure 1** organizes the hypothesis as several loops with different levels of evidence. Loop A links a lactate-rich environment to H3K18la and to a glycolytic or reparative macrophage program; the full lactate-to-lactylation-to-lactate sequence has not been demonstrated in EAMs. Changes in α-ketoglutarate availability or in the α-ketoglutarate/succinate ratio could modify dioxygenase activity, while fumarate can inhibit KDM5 enzymes and produce a training-like chromatin state in other systems (38). Loop C is intercellular. Macrophages induce ITGB3/αvβ3 in ectopic stromal cells and thereby increase stromal glycolysis and lactate production, but stable macrophage reprogramming by the returned lactate is still inferred (53). A related inflammatory circuit is better supported: NLRP3-activated macrophages release IL-1β, which promotes stromal-cell migration and lesion growth, and inhibition of this pathway suppresses disease (78). These loops need not all operate in every lesion. Their common feature is repeated signaling in a compartment that does not resolve normally.

Two qualifications are important. First, innate memory is not necessarily permanent. β-Glucan can reverse the chromatin state associated with endotoxin tolerance, showing that a stable program may be rewritten by a later stimulus (79). A pathogenic EAM state, if confirmed, could therefore remain therapeutically modifiable. Second, the relevant record may not be confined to mature cells in the peritoneal cavity. Trained immunity can arise in bone-marrow myeloid progenitors and alter the cells subsequently supplied to peripheral tissues (80). Whether endometriosis has such a central effect is unknown. Nevertheless, local and progenitor-level imprinting make different predictions about lesion recurrence after surgery and can be distinguished experimentally. We emphasize that these possibilities are not mutually exclusive: the proposed memory could be written locally *in situ* within the peritoneal niche, in resident and continuously recruited macrophages; maintained centrally in bone-marrow myeloid progenitors that supply the peritoneal compartment (central trained immunity); or both. A purely *in situ* imprint would predict that recurrence depends on residual or re-recruited local macrophages, whereas a central progenitor imprint would predict a systemic predisposition able to regenerate lesion-supporting macrophages even after apparently complete clearance. Whether the imprinting is exclusively peritoneal or also central is therefore an explicit, experimentally tractable question of this model rather than an assumption.

On current evidence, EAMs should be described as candidates for pathogenic training, not as definitively trained cells. Three findings support different parts of the proposal: macrophage states can be transferred in the tolerization model, EAM survival can depend on TET3, and histone lactylation can persist *in vivo*. These findings were obtained in different experimental contexts and do not yet form one causal chain. The decisive experiment would begin with a defined endometriotic niche signal, identify a persistent chromatin change in a human EAM subset, and show that this change carries a transferable disease-promoting function. Until that experiment is done, the hypothesis mainly serves to keep attention on how macrophage behavior changes over time, rather than treating lesion size or a metabolite concentration as a sufficient proxy.

## Lessons from chronic disease: maladaptive trained immunity

Experimental atherosclerosis provides a precedent for persistent myeloid reprogramming after a sterile metabolic exposure. In hypercholesterolemic mice, a short period of Western-diet feeding reprograms bone-marrow progenitors through the NLRP3 inflammasome. Hyperresponsive monocytes continue to be produced for weeks after the diet and systemic inflammation have subsided (81). The inducing exposure is transient, but the altered response is maintained in the progenitor compartment. This example does not prove an equivalent process in endometriosis. It does, however, establish biological precedent for a metabolically conditioned environment creating a disease-promoting innate program that outlasts the original stimulus.

There are also useful cellular parallels. Atherosclerotic plaques and endometriotic lesions are both sterile, chronically inflamed environments that recruit circulating monocytes. Their macrophages accumulate different forms of tissue-derived cargo—oxidized lipids in plaques and iron or heme in the peritoneal cavity—while being expected to clear damaged material. Trained immunity is now implicated in human inflammatory disorders and is not restricted to mouse models (9, 75). Tumors offer a second comparison: tumor-associated macrophages commonly adopt immunosuppressive and tissue-repair programs, and the same functional bias has been noted in EAMs (17). These analogies support plausibility, but they cannot substitute for evidence that EAM chromatin is stable or therapeutically reversible.

The main benefit of these comparisons is methodological. Atherosclerosis studies have already used adoptive transfer, progenitor lineage tracing, and inflammasome genetics to separate cell-intrinsic memory from continued environmental stimulation. Similar designs could determine whether an EAM phenotype survives removal from the lesion niche and whether it originates locally or in bone marrow. Endometriosis also introduces a complication absent from atherosclerotic plaques: its chemical and hormonal environment changes with the menstrual cycle. A macrophage program may therefore be reinforced in pulses rather than accumulated steadily.

## Menstrual cyclicity as a repeated input to macrophage state

The endometriotic niche is not a constant training environment. Eutopic endometrium and ectopic implants respond to ovarian steroids throughout the menstrual cycle, and macrophage abundance and phenotype in the reproductive tract change with the same rhythm. Predominantly M2-like cells increase during the secretory and menstrual phases (82). Retrograde menstruation is likewise episodic. Erythrocytes, heme, iron, cellular debris, and inflammatory mediators therefore enter the peritoneal cavity in recurrent waves. EAM studies should therefore model repeated pulses of exposure and should not infer steady stimulation from a single sampling point.

Evidence for estrogen effects is strongest at the level of macrophage recruitment and neuroimmune signaling. Lesional macrophages express ERα and ERβ, and Greaves et al. showed that 17β-estradiol increases recruitment and macrophage–nerve crosstalk through CSF1 and neurotrophin pathways (83). Estrogen can also amplify aromatase and PGE_2_ signaling in lesions. These findings make cyclical modulation of macrophages plausible, but no study has shown that an estrogen peak leaves a persistent macrophage chromatin mark. Progesterone is unlikely to provide a simple opposing signal: endometriotic tissue is frequently progesterone-resistant, and macrophages express little progesterone receptor, making most of its effects indirect (82). The existing data therefore support an estrogen-biased environment, not yet an estrogen-driven macrophage memory. Inflammation and sex-steroid signaling are bidirectionally linked in endometriosis: estradiol can promote inflammatory signaling, whereas excessive inflammation can alter sex-steroid receptor expression and aromatase activity (84). We therefore hypothesize that macrophage-derived cytokines within the peritoneal niche could help reinforce, rather than independently cause, progesterone-resistant stromal states. Conversely, impaired progesterone responsiveness could weaken anti-inflammatory restraint and prolong macrophage conditioning. Direct evidence for this macrophage–stromal feedback loop is not yet available.

Whether EAM chromatin changes with menstrual phase is unknown. One way to address the question would be to collect phase-matched samples and compare the same macrophage subsets for lactylation, methylation, metabolic activity, and transcription. Repeated sampling or an appropriate cyclical model would then show whether a mark is reinforced after estrogen exposure or lost during steroid withdrawal. Such data would also clarify treatment effects. Macrophage marks could be compared before and during hormonal suppression, and an intervention could be tested at different cycle stages. Until these experiments are performed, menstrual phase should be recorded as a biological variable and claims of a stable memory should not be based on a single sampling point.

## Therapeutic questions raised by the memory hypothesis

Clinical relevance depends on whether a retained EAM state can still be changed after it has formed. Blocking a short-lived upstream signal may be insufficient if the macrophage has already acquired a stable program. Current treatment reduces symptoms and lesion activity, but it has not been shown to remove immune states that could be relevant to persistence or recurrence (6). **Table 1** therefore separates candidate interventions according to what they would alter: the metabolic conditions under which a state is induced, the chromatin machinery that may retain it, or the macrophage phenotype after induction. Reversal of endotoxin tolerance by a later stimulus shows that an innate program is not necessarily permanent (79). Whether a naturally acquired EAM state can be rewritten safely remains an open experimental question. No clinical treatment has yet been shown to erase EAM memory. Nevertheless, three disease-specific preclinical observations support different components of therapeutic tractability: dichloroacetate lowers lactate and constrains disease in a mouse model (31); Bobcat339 degrades TET3 and reduces disease in tested endometriosis models (12); and experimentally imposed, IL-10-dependent macrophage tolerance limits disease (11). These observations address lesion metabolism, a TET3-dependent macrophage vulnerability, and the reversibility of an experimentally imposed macrophage state, respectively; none demonstrates durable erasure of a naturally acquired metabolic–epigenetic memory. **Table 1** ranks these observations together with less direct strategies by both endometriosis-specific evidence and relation to the proposed memory mechanism.

**Table 1:** Layered therapeutic strategies targeting metabolic–epigenetic coupling in endometriosis-associated macrophages, ranked by endometriosis-specific evidence.

Intervention layer	Representative agent/approach	Molecular target and mechanism	Evidence in endometriosis and relation to memory	Delivery/safety constraint	Key refs
Metabolic-cue withdrawal	Dichloroacetate (DCA)	PDK inhibition; lowers lactate and glycolytic flux	Direct metabolic evidence (mouse model); effect on EAM memory untested	Potential peripheral neuropathy with prolonged exposure; long-term systemic safety requires evaluation.	(31)
Metabolic-cue withdrawal	Lactate-lactylation pathway modulation	Reduce lactate availability or LDHA/EP300-linked lactylation signaling	Extrapolated	EAM-specific validation needed; avoid disrupting reparative programs	(10)
Epigenetic-record erasure	Bobcat339-mediated TET3 degradation	VHL-dependent TET3 degradation; preferentially depletes TET3-high pathogenic macrophages in preclinical models	Direct TET3 evidence (preclinical endometriosis); durability untested	Selectivity/long-term safety unknown	(12)
Trained-state reprogramming	Low-dose LPS tolerization/macrophage transfer	Installs an IL-10, disease-limiting memory	Direct state-reprogramming evidence (mouse model); not natural-memory erasure	Translational route undefined	(11)
Trained-state reprogramming	β-glucan	Reverses LPS-tolerance-associated epigenetic state in trained-immunity models	Extrapolated	Directionality risk; not a direct endometriosis therapy	(79)
Trained-state reprogramming	4-octyl itaconate/itaconate axis	SDH inhibition and NRF2 activation through KEAP1 alkylation	Extrapolated	Endometriosis-specific validation needed	(56, 57)
Downstream mediator	EP receptor antagonist/lipoxin A4	Dampen the PGE_2_–estrogen amplifying loop	Partial (endometriosis models)	Symptomatic; upstream memory left intact	(86, 87)
CSF1R/KIT signaling	Pexidartinib (CSF1R/KIT)	Suppresses CSF1R/KIT-linked inflammatory signaling and viability in 12Z endometriotic epithelial cells	Partial/*in vitro*	Macrophage-targeted efficacy remains untested; host-defense and tissue-repair impact	(89)
Macrophage recruitment	CCL2–CCR2 modulation	Reduces recruited monocytes; lesion efficacy was not demonstrated	Direct counterevidence (mouse model)	Origin-specific macrophage effects; indiscriminate blockade may be ineffective	(88)

Evidence tiers: Direct = the intervention or mechanism was studied in an endometriosis model; this does not by itself prove durable erasure of EAM memory or clinical efficacy. Partial = partial, *in vitro*, or ex vivo evidence in endometriosis. Extrapolated = evidence from trained-immunity or other disease models awaiting endometriosis-specific validation. The table lists interventions mechanistically related to the proposed EAM memory framework and excludes conventional hormonal or surgical therapy.

Targeting the metabolic input is the most immediately tractable strategy, because the enzymes of glycolysis are well-characterized drug targets. Lowering lactate could reduce substrate availability for histone lactylation: the pyruvate dehydrogenase kinase inhibitor dichloroacetate normalizes lesion metabolism, reduces peritoneal lactate, and constrains disease in preclinical endometriosis models (31). The logic extends beyond a single agent. Pharmacological inhibition of lactate production or of the lactylation reaction itself blocks the acquisition of trained immunity, and genetic variation in LDHA and the lactylation writer EP300 modulates the strength of that memory, identifying both as rational targets for interrupting the lactate-to-lactylation arm (10). Whether such interventions alter EAM lactylation and lesion persistence *in vivo* remains to be tested.

Targeting the epigenetic record addresses the durability that metabolic intervention alone may not overcome, and endometriosis now has disease-specific preclinical evidence that a TET3-dependent macrophage vulnerability is targetable. Because pathogenic TET3-overexpressing macrophages depend on elevated TET3 for survival, the synthetic cytosine derivative Bobcat339 triggers VHL-dependent degradation of TET3, preferentially targets TET3-high macrophages in the tested models, and strongly inhibits endometriosis progression in mice (12). This result is important beyond its specific target, because it demonstrates that a TET3-dependent vulnerability induced by inflammatory niche signals can be exploited therapeutically; it has also been discussed as a concept strategy for endometriosis treatment (85). The same principle motivates interest in more general writers, erasers, and readers implicated in macrophage memory, but HDAC inhibitors, BET inhibitors, and lactylation-blocking strategies should be treated as extrapolated rather than endometriosis-validated. Their application to EAMs would require selectivity to avoid disrupting protective epigenetic programs.

A third approach would attempt to replace the trained phenotype rather than target one enzyme. Repeated low-dose endotoxin produces an interleukin-10-dependent, disease-limiting state in peritoneal macrophages, and transfer of those cells suppresses lesion growth (11). This result shows that an opposing innate program can alter disease, but low-dose endotoxin is not a practical therapy. Likewise, β-glucan can reverse endotoxin-tolerance chromatin, yet it induces a different trained state and should not be interpreted as an endometriosis treatment (79). Metabolite-derived compounds may offer more tractable ways to steer macrophages. Itaconate and 4-octyl itaconate inhibit succinate dehydrogenase and activate NRF2 through KEAP1 alkylation, producing anti-inflammatory effects in macrophage models (56, 57). Downstream mediators can also be blocked: selective PGE2 receptor antagonists act on both endometriotic stromal cells and peritoneal macrophages (86), and lipoxin A4 limits lesion progression while reducing PGE2 and estrogen signaling (87). None of these approaches has yet shown that a naturally acquired EAM memory has been durably reset.

Delivery is the practical constraint that determines whether any of these strategies can be realized safely, because the relevant enzymes and metabolic pathways operate throughout the body. Macrophage-directed approaches might confine intervention to pathogenic subsets, but origin-specific functions make indiscriminate depletion or recruitment blockade risky. In a mouse model, a function-blocking CCR2 antibody reduced recruited monocytes without significantly reducing lesion number, underscoring that altered recruitment is not equivalent to therapeutic efficacy and that resident and monocyte-derived macrophages can have opposing effects (88). Targeting CSF1R/KIT signaling is another candidate route, but macrophage-directed efficacy remains unproven. CSF1R is detected in lesion stroma and macrophages, whereas pexidartinib has so far been shown to suppress inflammatory signaling and viability in 12Z endometriotic epithelial cells rather than to deplete lesion macrophages (89). This pathway is consistent with the CSF1-dependent macrophage populations found in ovarian endometriosis (90). The convergence of macrophage-selective delivery with a memory-directed epigenetic or metabolic payload is therefore a testable translational direction, not an established treatment strategy.

Endometriosis is a multifactorial disease, and macrophage memory is advanced here as one tractable node within a broader immune network rather than as its sole driver (15). Humoral immunity is also altered in endometriosis, with reports of increased or activated B-cell populations and excessive autoantibody production; however, study heterogeneity and limited mechanistic data prevent assignment of a uniform causal role (91). Macrophage-derived cytokines, tissue injury, and antigen presentation could shape B-cell activation, whereas antibodies or immune complexes could in turn alter Fc-receptor signaling in macrophages, but these reciprocal links have not been demonstrated as drivers of EAM memory. Clinical experience also cautions against assuming that cytokine neutralization will reverse the proposed state. In a small, randomized placebo-controlled trial, infliximab did not improve pain associated with deep endometriosis (92), and a Cochrane review found insufficient evidence to support anti-TNF-α therapy for endometriosis-associated pelvic pain (93). Thus, B-cell/antibody abnormalities and cytokine-directed interventions provide relevant immune context, but neither constitutes a validated extension or treatment of the macrophage-memory model.

Among the candidate targets, TET3 has unusually direct support in endometriosis. The same disease context contains the proposed upstream inflammatory signals, a TET3-high macrophage population with a survival dependency, and a degrader that reduces disease in experimental models. The evidence for lactylation inhibitors, itaconate derivatives, or broader trained-immunity interventions is less direct and comes mainly from other macrophage systems. Before clinical translation, these agents need to be tested in lesion-specific macrophage subsets and across the changing hormonal conditions of the peritoneal cavity. A response observed at one time point or in one lesion type should not be assumed to generalize to ovarian, peritoneal, and deep-infiltrating disease.

## Clinical translation and candidate biomarkers

The metabolites in this model are relevant not only as drug targets but also as possible biomarkers. Noninvasive diagnosis of endometriosis remains difficult. Current guidance favors imaging, with laparoscopy considered when imaging is negative and empirical treatment is unsuccessful or inappropriate (94). Local metabolites may enter the circulation, so peritoneal-fluid chemistry could, in principle, inform a blood-based assay (30). Lactate is an obvious starting point because it is repeatedly elevated in peritoneal fluid and lies at the center of the proposed macrophage-memory mechanism. Independent metabolomic series also report succinate, pyruvate, and other glycolytic intermediates (47). These molecules are not specific to endometriosis, however. Their diagnostic value will depend on external validation, multianalyte combinations, and careful control for systemic metabolic conditions. A causal relationship with EAM memory has not yet been established. In particular, bulk peritoneal-fluid or circulating lactate and succinate reflect the aggregate metabolism of many cell types and of recurrent bleeding, so on their own they lack the diagnostic specificity needed either to identify endometriosis or to report on macrophage memory. A memory-based biomarker would therefore have to move beyond bulk metabolite concentrations to cell-specific epigenetic signatures—for example, lactylation- or TET3-associated marks measured in sorted or single-cell-resolved peritoneal or circulating macrophage subsets—that read out the pathogenic program itself rather than the ambient metabolic milieu.

The more distinctive opportunity is stratification. If pathogenic macrophage memory contributes to lesion persistence, then a readout of that memory, such as a lactylation- or TET3-associated epigenetic signature in circulating or peritoneal-fluid macrophages, could identify the patients in whom the memory is most entrenched and who might therefore benefit most from a memory-directed intervention. Such a marker could also offer a way to judge whether a therapy has altered the pathogenic program rather than merely suppressing symptoms, supplying a recurrence-relevant endpoint that current biomarkers lack. This remains aspirational: no epigenetic biomarker of EAMs has yet been validated, and moving from a mechanistically attractive candidate to a clinically deployable assay will require the single-cell and spatial methods discussed below.

Therapeutic and diagnostic development therefore share a measurement problem. Studies must determine whether the metabolites proposed as inputs and the chromatin marks proposed as records occur together in the same macrophage populations. If they do, one biological axis could yield a circulating analyte, a tissue-based stratification marker, and a pharmacodynamic readout. If they do not, biomarker discovery should remain separate from the memory hypothesis rather than being forced into it.

## Testing the hypothesis: limitations and next experiments

The evidence assembled here supports a working hypothesis, but not an end-to-end mechanism. No study has yet followed a specified niche metabolite into a persistent epigenetic mark and then into a transferable pathogenic function in human EAMs. Many of the biochemical links discussed in this article were established in cultured macrophages or in diseases other than endometriosis. Applying them to the peritoneal niche is reasonable but remains an inference. The immediate priority is therefore causal testing: changing a candidate metabolite should alter a defined macrophage chromatin mark, and manipulating that mark should change macrophage behavior and disease. Such experiments must be interpreted in the context of an endometriosis pathophysiology that is itself incompletely resolved (95).

Resolving this will require methods matched to the problem’s spatial and temporal structure. Bulk metabolomic surveys describe average peritoneal fluid rather than the metabolites a macrophage experiences at a lesion’s surface, and bulk epigenomic assays obscure the heterogeneity that single-cell atlases have made unmistakable (16). Single-cell and spatial multi-omics that jointly resolve metabolism, chromatin state, and transcription *in situ* are needed to test whether specific metabolite gradients align with specific epigenetic marks in the pathogenic subsets identified by reference atlases (5). Early spatial studies of lesions, which resolve epithelium-macrophage crosstalk (19) and fibroblast-immune niches (96), show that the requisite tools now exist even if they have not yet been turned on the metabolic-epigenetic axis. Lineage-tracing and adoptive-transfer designs will be required to distinguish an imprinted, cell-intrinsic memory from a continuously instructed state, and to determine whether the relevant reprogramming occurs locally, in bone-marrow progenitors, or both.

Several features of endometriosis need to be built into those experiments. The disease is cyclical and estrogen-dependent, so both niche composition and macrophage exposure are likely to vary with menstrual phase. Longitudinal or phase-matched sampling will be needed to determine whether steroid rhythms reinforce or erase candidate marks (82, 83). Lesion type is another source of biological variation. Peritoneal, ovarian, and deep-infiltrating disease occupy different tissue environments and need not contain the same macrophage states. Metabolite action is also conditional: succinate and oxysterols can produce opposing responses at different concentrations or in different cellular contexts. A signal associated with memory in one lesion type may therefore be neutral or even protective in another.

Safety is likely to be the main translational constraint. Systemic alteration of macrophage chromatin could interfere with antimicrobial defense, wound healing, or tumor surveillance. In a benign disorder, prolonged exposure to an epigenetic drug would require strong evidence that the pathogenic subset is targeted while protective macrophage functions are preserved. Recurrence is a second unresolved issue. No memory-directed treatment has yet been evaluated for prevention of recurrent disease. Masferrer-Ferragutcasas and colleagues have argued that recurrence should be investigated as a biological process rather than accepted as an unavoidable outcome (97). The present hypothesis suggests a specific test: after lesion removal, researchers could determine whether a local macrophage or bone-marrow progenitor state remains and whether its persistence predicts subsequent lesions.

The proposal yields several separable tests. After withdrawal from the lesion environment, an imprinted macrophage should retain its phenotype for a defined period. Transfer of the cell, or of progeny derived from an affected precursor, should carry part of that phenotype into a new setting. Manipulating the suspected metabolic input should change acquisition of the state, whereas targeting the associated chromatin mechanism should change its maintenance or recall. Finally, a directed intervention should reverse the phenotype without broadly disabling normal macrophage function. At present, individual observations support parts of this scheme in different systems. Failure at any of these steps would narrow or refute the proposed memory mechanism in EAMs. **Table 2** summarizes these predictions alongside the current level of supporting evidence and the key experiment required to test each.

**Table 2:** Falsifiable predictions of the macrophage metabolic–epigenetic memory model, the current level of supporting evidence, and the key experiment required to test each prediction.

Prediction	Rationale within the model	Current supporting evidence (tier)	Key experiment to confirm or refute
Persistence after stimulus withdrawal	A chromatin mark can outlast the metabolite that licensed it, so the phenotype should remain after the inducing niche signal is removed.	Histone lactylation persists for months *in vivo* (Extrapolated, 10); TET3-high pathogenic macrophages were identified in endometriosis and exhibit TET3-dependent survival (Direct endometriosis-model evidence, 12).	Track EAM phenotype and candidate marks at defined intervals after removal from the lesion niche.
Transferability	A cell-intrinsic record, not continuous instruction, should carry the phenotype into a naive host.	Tolerized peritoneal macrophages transfer disease protection to untreated recipients in mice (Direct, 11).	Adoptive transfer and progenitor lineage-tracing to test transfer of the pathogenic (not only protective) state.
Metabolic dependence	Niche metabolites act as substrates or cofactors for chromatin-modifying enzymes.	TGF-β1-driven lactate output (Partial, 32); dichloroacetate lowers lactate and constrains disease (Direct, 31); LDHA/EP300 variation tunes trained memory (Extrapolated, 10).	Manipulate a defined metabolite (e.g., lactate) and measure a defined EAM chromatin mark and function.
Chromatin-associated maintenance	Persistent marks (H3K18la; TET/JmjC-regulated methylation states) maintain the disease-promoting state.	H3K18la at reparative-gene promoters/enhancers (Extrapolated, 8, 67); TET3-dependent program in EAMs (Direct, 12).	Single-cell and spatial multi-omics linking marks to transcription in pathogenic subsets *in situ*.
Reversibility	Innate memory is in principle rewritable, which defines a therapeutic opportunity.	β-glucan reverses endotoxin-tolerance chromatin (Extrapolated, 79); Bobcat339/TET3 degradation reduces lesion burden, and LPS tolerization installs an opposing disease-limiting macrophage state (Direct endometriosis-model evidence, 11, 12).	Test whether a memory-directed intervention yields durable loss of lesion-supporting function versus transient suppression.

## Conclusions

We propose that some macrophages in endometriosis may retain a disease-supporting imprint instead of merely adopting a transient polarization state. In this model, repeated metabolic, inflammatory, and endocrine signals from the niche alter macrophage chromatin and prolong phenotypes that favor lesion survival. Glycolysis, histone lactylation, and the TET3-dependent macrophage program provide separate pieces of evidence for this idea, but they have not yet been connected in one causal experiment. The model is therefore deliberately provisional. Its value is that it defines measurable features of memory and identifies metabolites and chromatin regulators that could serve as biomarkers or therapeutic targets.

Disease-specific evidence is currently concentrated in a small number of studies, particularly the macrophage training/tolerization experiments and the TET3 work. The proposed metabolite-to-chromatin-to-function sequence still needs to be demonstrated in human tissue. Doing so will require models that preserve the cyclical, estrogen-responsive, and spatially organized character of the lesion niche. Single-cell and spatial approaches should measure metabolic state, chromatin, and transcription in matched macrophage subsets, while lineage tracing and transfer experiments should test whether the phenotype is cell-intrinsic and how long it lasts.

If those studies confirm persistence, metabolic dependence, transferability, and reversibility, macrophage-selective interventions could then be evaluated against lesion persistence and recurrence. The relevant durable target may prove to be neither a single metabolite nor the visible lesion alone, but the macrophage program repeatedly reinforced by the endometriotic microenvironment.

## Data Availability

The original contributions presented in the study are included in the article/supplementary material, further inquiries can be directed to the corresponding author/s.
